# Maternal Self-Construal, Maternal Socialization of Emotions and Child Emotion Regulation in a Sample of Romanian Mother-Toddler Dyads

**DOI:** 10.3389/fpsyg.2018.02680

**Published:** 2019-01-11

**Authors:** Oana Benga, Georgiana Susa-Erdogan, Wolfgang Friedlmeier, Feyza Corapci, Mara Romonti

**Affiliations:** ^1^Department of Psychology, Babeş-Bolyai University, Cluj-Napoca, Romania; ^2^Department of Psychology, Grand Valley State University, Grand Rapids, MI, United States; ^3^Department of Psychology, Boğaziçi University, Istanbul, Turkey

**Keywords:** maternal self-construal, maternal socialization, child emotion regulation, social regulation of emotion, toddlers

## Abstract

Over the recent years, there is growing recognition of the social and cultural regulatory processes that act upon individual emotions. The adult-to-child social regulation of emotion is even more relevant, given the development of child self-regulatory abilities during early years. Although it is acknowledged that parental regulatory attempts to their children’s emotional expressions are influenced by cultural models, relatively little is known about the specific relationship between parental cultural models and socialization practices that foster emotion self-regulation, particularly in the case of toddlers. Therefore, in the present study, our first aim was to examine, in a Romanian sample of mother-toddler dyads, the relationships between maternal cultural model of self and maternal regulatory attempts targeting toddlers’ emotions during a delay of gratification task, while controlling for maternal perceptions of child individual characteristics, namely temperament. The second aim was to analyze, within the delay of gratification task, the relations between maternal regulatory attempts, child regulatory strategies and child affect expression, as the outcome of emotion regulation. Results showed that mothers scored higher for Independence as compared to Interdependence dimensions of self-construal. Also, the multidimensional analysis of self-construal revealed that Autonomy/Assertiveness scores were significantly higher than Relational Interdependent scores. Moreover, different dimensions of Independence predicted different maternal regulatory strategies employed during the delay of gratification task. This pattern of results suggests that maternal representations of an independent self, evidenced in our sample, are reflected in regulatory practices, aimed to develop primary control in the toddler. Moreover, our data revealed several significant associations between maternal regulatory strategies and child regulatory strategies expressed during the delay of gratification task. Finally, we demonstrated that child self-regulation mediated the relation between maternal regulatory attempts and child expression of affect during this task.

## Introduction

The prevalent, traditional approach to emotion regulation stems from the individual’s perspective. As such, regulation of emotion is essentially self-regulation, consisting in “behaviors, skills, and strategies, that serves to modulate, inhibit, and enhance emotional experiences to accomplish one’s goals” (Calkins and Hill, 2007, p. 229). However, over the recent years there has been growing recognition of the social and cultural regulatory processes that act upon individual emotions. Social regulation of emotion refers to the act of modifying someone’s emotional experience by the social environment, and in particular, by closely related persons (Reeck et al., 2016). The cultural view on emotion regulation adds to this perspective the idea that emotional experiences are modulated in culturally congruent and valued ways, by (cultural) models of self and relating (Mesquita et al., 2014).

### Social Regulation of Emotions in Early Development

From a developmental perspective, the adult-to-child social regulation of emotion is critical, since it not only improves the current emotional state of the child, but also contributes to the building of her/his self-regulatory abilities (Kopp, 1989). In the first 3 years of life, emotion regulation emerges from experiences within the parent-child relationship (Kiel and Kalomiris, 2015). Yet, children younger than three frequently rely on adult external support, to respond to emotionally saturated situations in a self-regulated manner (Grolnick et al., 1996). The tension between toddler’s increasing need for autonomy and her/his emergent self-control abilities, brings significant challenges for both parent and child, in such situations. Hence, a main socialization goal of caregivers is that of supporting their toddlers’ ability to control impulses and regulate child emotional expressions (Feldman et al., 2006).

Delay of gratification, that is, the ability to modulate emotions and behaviors while resisting to immediate temptations, such as waiting for a reward or delaying to play with an attractive toy, is considered a key self-regulatory ability that emerges during toddlerhood (Cole et al., 2011). Existing studies have shown that mothers’ use of distraction, alone or coupled with limit-setting responses, concurrently and longitudinally predicted delay of gratification ability (LeCuyer and Houck, 2006). When maternal and child behaviors were examined concurrently, mothers’ verbal distraction during a waiting task was positively related to toddlers’ verbal distraction, but also to higher levels of venting/aggression (Mirabile et al., 2009), and emotional distress (Grolnick et al., 1998). However, all these results come from Western, in particular United States, samples, bringing up the question of their generalizability to other cultural niches.

### Cultural Models of the Self

Although it is acknowledged that parental regulatory attempts are influenced by cultural models (Keller et al., 2004), relatively little is known about the specific ways through which culture infuses socialization practices, in order to foster emotion self-regulation, particularly in the case of toddlers. Recent approaches have theorized that self-construal, a construct that reflects the cultural differences in the self-concept, regarding how separate or connected individuals see themselves in relation to others (Markus and Kitayama, 1991), underlies cultural patterns in caregiving responses (Rothbaum and Wang, 2010). Since contexts provide different opportunities to experience the self (Coskan et al., 2016), self-construal is expected to vary across cultures.

In Western/North American urban communities, individuals perceive themselves as separate from others, prioritizing personal agency and goals. Thus, an independent view of the self, as separate from others, autonomous and stable, is rather normative. In contrast, in non-Western societies, there is an emphasis on interconnectedness, interdependence and minimizing individuality (Markus and Kitayama, 1991; Singelis, 1994). Accordingly, an interdependent self-construal, that is, closely connected to others, flexible and variable, is characteristic in this case (and largely studied in, though not restricted to East Asian contexts).

Self-construal is an individual-level construct, considered to reflect the cultural orientation of a certain society toward individualism versus collectivism. In this framework, individualistic cultures promote an independent self-construal, while collectivist ones promote an interdependent self-construal (Cross et al., 2011). Other researchers (Kagitcibasi, 2007; Vignoles et al., 2016) have called into question this binary conceptualization of the self, as well as its isomorphism with nation-level constructs, like individualism-collectivism (Hofstede et al., 2010).

In this sense, Kagitcibasi (2007) showed that, with economically driven socio-demographic changes, educated urban/suburban families in collectivist countries (e.g., India, China, Japan, Turkey; see also Costa Rica, Keller et al., 2004) shift toward autonomy, while still valuing relational affiliation. The self, in these contexts, becomes an autonomous-related one, organizing autonomous acts around relationships with close others and displaying autonomy granting, along with warmth and order-setting control. Consequently, such cultural niches are thought to promote a third model of the self-construal, that is a mixture of independence and interdependence, namely, the emotionally (or psychologically) interdependent self-construal (Kagitcibasi, 2007).

Like other collectivist societies undergoing significant sociodemographic changes, Romania is considered representative for the emotional interdependence model, given the major shifts toward democracy, market economy, urbanization, and globalization, over the post-communist period (since 1990), augmented after having joined the European Union in the last decade (Gavreliuc and Ciobotă, 2013; Corapci et al., 2017).

### Parental Cultural Orientation and Child Self-Regulation

Evidence regarding the impact of different parental self-conceptualizations on emerging child self-regulation is rather indirect, since most often self-construal is not evaluated in relationship to parent and/or child variables; in addition, such evidence is rather limited and challenging (e.g., Jaramillo et al., 2017).

Most approaches circumscribe the dichotomy between independence- and interdependence-promoting cultures, while only few attempts address the emotional interdependence-promoting cultural contexts. On the one hand, in independence-promoting cultures, an important aim for early childhood development is the ability to interact independently with the world, based on ones’ own goals and intentions. Consequently, self- and emotional control are central socialization goals, whereas parental strategies, characterized by less directiveness and more autonomy granting, are thought to have beneficial effects on child regulatory development (Rothbaum and Wang, 2010; Corapci et al., 2017). On the other hand, when comparing the proximal infant parenting style, characteristic for interdependence-promoting cultures, with the more distal parenting style of independence-promoting ones, Keller et al. (2006) emphasized that the first was associated with earlier emergence of self-regulation in toddlerhood. Similarly, Lamm et al. (2017) showed that better delay of gratification abilities, and better emotional control at 4 years of age, were predicted by the responsive control style of rural Cameroonian mothers, with a strong interdependent orientation, as compared to the more observant style of urban German mothers, who have a strong orientation toward independence.

In addition, Keller et al. (2004) evidenced that children of mothers with an emotional interdependence orientation (from highly educated families in Costa Rica) fall between those from interdependent (Cameroonian Nso) and independent (middle class Greek) cultural niches, in terms of the developmental timeline of self-regulation. However, studies on self- and emotion regulation, and in particular on their temporal dynamics, in emotionally interdependent cultural contexts, are rather scarce.

Not only the timing of self-regulation development in toddlerhood remains a critical issue in the light of existing evidence, but also the cultural significance attributed to such abilities. One interesting claim (Trommsdorff, 2009) is that self-regulation might be understood and promoted either as motivated action, aimed to achieve autonomy by exerting influence over others, in independence-promoting cultures, or as interpersonal regulation, in order to adjust the self to the expectations of others, in interdependent contexts. In the same vein, it was suggested that parents from independence-promoting societies inculcate in their children primary control, which means the world is changeable, whereas the self is something fixed, so the world may be transformed to better adapt to the individual. On the other hand, parents from interdependence-promoting societies socialize their children toward secondary control, which means the world is fixed and the individual self is changeable (Rothbaum and Wang, 2010; Jaramillo et al., 2017).

In terms of this dichotomy, there still remains the question on the meaning assigned to child self-regulatory abilities in emotionally interdependent cultural niches, which promote both independence and interdependence.

### Parental Cultural Orientation and Child Emotion Socialization

If emotions are regulated to improve their match with the prevalent models of self and relating (Mesquita et al., 2014), we should expect parallel distinctions between independent and interdependent cultural orientations, in this respect. Evidence confirms that participants from independent cultural contexts experience and value positively more socially disengaging emotions, like pride, anger, and irritation, while those from interdependent ones report and also favor more socially engaging emotions, like guilt, shame, and closeness (Kitayama et al., 2006). In terms of social regulation of emotion, independence-oriented parents encourage the expression of socially disengaging emotions, like anger or dissatisfaction (Heikamp et al., 2013), while providing greater situational variability that increases the probability of more diverse positive as well as negative emotional experiences (Rothbaum et al., 2000). On the contrary, interdependence-oriented parents downplay the negative disengaging emotions, by challenging the interpretation of a situation as frustrating or distressing, and by supporting child acceptance (Heikamp et al., 2013). Meanwhile, these parents structure the environment to provide a safe and stable setting, thus decreasing the probability of emotional escalation (Rothbaum et al., 2000).

As regarding parents with the third orientation, namely, the emotional interdependence, their responses toward child’s negative disengaging emotions entail more subtle ways of emotion disapproval, along with warmth and reasoning, for example by referring to social norms and empathic understanding of others (Cheah and Rubin, 2004; Raval et al., 2013). In the meantime, these parents highly endorse problem-focused skills and strategies, which is consistent with the value they put on child autonomy. This mixture of comforting, reasoning and problem-focused responses is considered to reflect the combined independent and interdependent (autonomy and relatedness) orientations, the hallmark of emotional interdependence.

### Cultural Context of Urban, Middle-Class Romanian Families

While evidence is accumulating regarding cross-cultural differences in self-regulation, there is still little information about cultures outside the standard individualism versus collectivism dichotomy. Romania provides an interesting case study, from this perspective. According to Hofstede’s scores (Hofstede et al., 2010), it is a rather collectivistic country, high in power distance and uncertainty avoidance, low in indulgence, relatively feminine and with a predominant long-term orientation. However, the sociodemographic changes undergone over the last decades are expected to be associated with a decrease in collectivistic socialization values and a corresponding increase in the individualistic ones (Greenfield, 2009). Studies at the societal level confirm this prediction, reporting a certain ambivalence, reflected in the presence of both individualism and collectivism (Gavreliuc, 2011). A more fine-grained analysis in terms of different regional profiles suggests that Transylvania (the geographical region of our sample) has the highest scores on individualism, above the country level (Neculăesei and Tãtăruşanu, 2008) – as well as lower uncertainty avoidance, coupled with higher indulgence. Thus, we expect a more pronounced individualistic orientation in this region, within the ambivalent pattern.

On their turn, societal changes seem to be reflected in the individual self-construal. Self-descriptions of young Romanians also have an ambivalent structure, at the same time independent and interdependent (Gavreliuc and Ciobotă, 2013; see also Shulruf et al., 2011). One comparative study shows that Romanian students perceive themselves as both more independent and more interdependent than United States students (Moza et al., 2015). However, another study with university students, reported by Moza and Gavreliuc (2017 unpublished), evidences that the level of independence in the self-construal of the Romanian sample is higher than that of the United States sample, while the level of interdependence is similar with that of the Japanese sample. These data converge into suggesting that middle-class, educated Romanians promote a rather emotionally interdependent (autonomous-related) self-construal. Yet, the independence-interdependence balance in the self-construal is not rigidly set, therefore it deserves further exploration, particularly in relationship with family dynamics and child socialization.

Family relationships (extended and kin family interdependence) and childrearing are highly valued in Romania (Robila and Krishnakumar, 2004). Still, the few existing data on young children socialization reveal that Romanian mothers report both relational and autonomy goals in socializing young children (with a slight preference for the last), in line with the emotional interdependence model (Mone et al., 2014). In terms of emotion regulation, Romanian mothers of preschoolers emphasize teaching their children to down regulate anger and sadness, while in the case of happiness, they tend to match their children’s expressions (Denham et al., 2004). More recently, Corapci et al. (2017) also found evidence for the emotional interdependence model in Romanian mothers, when assessing interview responses targeting toddler socialization in emotionally charged situations.

### Present Study

The present paper aimed to analyze the socialization of emotions in toddlerhood, by exploring maternal regulatory attempts on child emotional displays, within a delay of gratification task. Given the weight cultural context may put on these processes, as recent approaches suggest, we investigated maternal regulatory behaviors in relationship to maternal cultural models of self. Our theory-guided selection of sample was based on Kagitcibasi’s (2007) model of emotional interdependence, illustrated by several studies on the Romanian cultural context of the last two decades (e.g., Mone et al., 2014; Moza et al., 2015; Corapci et al., 2017).

The first aim of the current research was to examine the relationships between maternal self-construal dimensions and maternal regulatory attempts targeting toddlers’ emotions during a delay of gratification task, while controlling for maternal perceptions of child individual characteristics (namely, temperament), child gender and maternal education. The second aim was to analyze, within the delay of gratification task, the relations between maternal regulatory attempts, child regulatory strategies, and child affect expression, as a result of emotion regulation.

Regarding the first aim, based on the existing literature that supports a view of an emotionally interdependent self-construal in the younger, middle-class, educated Romanians, we predicted that Romanian mothers from our sample would equally use independent and interdependent self-descriptions. We also extended the exploration of the self-construal (Singelis, 1994; Hardin et al., 2004; Christopher et al., 2012), by including, along the higher-order dimensions of Independence and Interdependence, their subdimensions: for Independence — Autonomy/Assertiveness, Individualism, Behavioral Consistency, Primacy of Self; for Interdependence — Esteem for Group, Relational Interdependence. Accordingly, we expected that Autonomy/Assertiveness scores will be similar to Relational Interdependence ones.

We further explored the relations between all maternal self-construal dimensions/subdimensions and maternal regulatory strategies. In terms of specific predictions, based on previous literature (Rothbaum and Wang, 2010; Jaramillo et al., 2017), we expected that an emphasis on Independence (but also Individualism and Autonomy/Assertiveness) would be negatively related with strategies that focus on task-oriented control (like Removing Cookie) and positively associated with maternal strategies that stress agency and encourage children to engage in goal-directed behavior (Distraction). On the other hand, following Cheah and Rubin (2004) and Raval et al. (2013), we expected that an emphasis on Relational Interdependence will be positively associated with endorsement of warmth (e.g., Physical Comfort) and positive control strategies (e.g., Reasoning).

We examined the potentially confounding role of maternal perception of child temperament, given its possible association with maternal self-construal dimensions and maternal regulatory attempts, respectively. We based this assumption on data showing that, in both collectivistic and individualistic societies, parents more frequently use controlling strategies with children perceived as high in temperamental Negative Affectivity and Surgency, but less frequently with children perceived as high on temperamental Effortful Control (Cole et al., 2003; Xing et al., 2017).

Regarding the second aim, based on the developmental perspective of the adult-to-child social regulation of emotion in toddlerhood (Kopp, 1989; Reeck et al., 2016), we explored the directions (positive or negative) of the associations between maternal regulatory strategies, child regulatory strategies, and child affect, during the delay of gratification task. In addition, we predicted that child self-regulation will mediate the relation between maternal regulatory attempts and child expression of affect during the delay task.

## Materials and Methods

### Participants

Participants were recruited both through flyers distributed in childcare and play centers, and also through in-person invitations made by research assistants. Parents were invited to participate in the study through written information distributed by directors and childcare educators. Forty-five dyads signed the consent form initially, but only 40 dyads completed all measurements. Therefore, our final sample included 40 dyads (21 boys, age range = 18–32 months, *M*= 24.3, *SD*= 3.44). All children had no clinical diagnosis to indicate the presence of psychopathology as reported by educators and childcare psychologists. All participants were from Cluj-Napoca, the second most populous city in Romania, and one of the most important industrial/business, as well as academic/cultural centers in the country, and Transylvania region, respectively. Mean mother age (years) was 31.5 (*SD* = 3.2) and the majority (83%) were primiparous mothers. Ninety-three percent had at least the college degree, while 7% graduated a trade, technical, vocational, or business school. Moreover, 74.4% were employed and 93% married (while the other 7% were either single or divorced).

### Procedure

After they returned the informed consent, mothers and their toddlers were invited in the Developmental Psychology Laboratory, located in the University campus, for a one-time (per child) observational session, that is, a 4-min delay of gratification task. All experimenters were trained graduate psychology students. The task was video recorded with mother’s consent. Maternal and child regulatory attempts, as well as child emotional expressions, manifested during task, were later coded, using each dyad’s recording. At the end of the observational task, mothers were given questionnaires to report on family demographics, as well as child temperament. They were asked to complete questionnaires either in the laboratory or at home, in the last case being provided with a postage-paid, return addressed envelope.

### Measures

#### Maternal Self-Construal

Mothers’ self-construal was evaluated with the Self-Construal Scale (Singelis, 1994), consisting of two higher-order dimensions, Independence and Interdependence, each containing 15 items rated on a 7-point Likert scale (*1 = strongly disagree* to *7 = strongly agree*). Mean scores were computed for each dimension by dividing each dimension’s total score by the number of items. We also calculated (based on the multidimensional approach proposed by Hardin et al., 2004) the six self-construal subdimensions: Autonomy/Assertiveness (7 items e.g., “I can talk openly with a person whom I meet for the first time, even when this person is much older than I am”), Individualism (6 items e.g., “I enjoy being unique and different from others in many respects”), Behavioral Consistency (2 items e.g., “I act the same way no matter who I am with”), Primacy of Self (3 items e.g., “I try to do what is best for me, regardless of how that might affect others”), Esteem for Group (8 items e.g., “I have respect for the authority figures with whom I interact”) and Relational Interdependence (4 items, e.g., “I often have the feeling that my relationships with others are more important than my own accomplishments”). Cronbach’s alphas are presented in Table 1. Reliabilities reported by other studies are within the following ranges: Independence 0.68, Interdependence 0.86 (Romanian sample, Moza and Gavreliuc, 2017 unpublished); Independence 0.70–0.73, Interdependence 0.67–0.74, Autonomy/Assertiveness 0.41–0.47, Individualism 0.61–0.66, Behavioral Consistency 0.18–0.73, Primacy of Self 0.45–0.58, Esteem for Group 0.56–0.63 and Relational Interdependence 0.44–0.59 (multiple samples, Hardin, 2006, as shown by Christopher et al., 2012).

**Table 1:** Means, standard deviations and reliability scores for SCS.

Self-construal higher order
dimensions and subdimensions	*M*	*SD*	α
Independence	5.31	0.58	0.90
Interdependence	4.98	0.61	0.92
Autonomy/assertiveness	4.83	0.60	0.62
Individualism	5.86	0.61	0.54
Behavioral consistency	5.18	1.07	0.79
Primacy of self	4.91	0.86	0.99
Esteem for group	5.31	0.63	0.85
Relational interdependence	4.45	1.03	0.81

#### Maternal Perception of Toddler Temperament

Toddler temperament was assessed with the Early Childhood Behavior Questionnaire–Very Short Form (ECBQ–VSF; Putnam and Rothbart, 2006). Mothers completed the 36 items rated on a 7-point Likert scale (*1 = never* to *7 = always*) examining how their child reacts in different situations. This questionnaire is composed of three subscales: Surgency/Extraversion, which reflects positive emotional reactivity (smiling, pleasure), Negative Affectivity, referring to negative emotional reactivity (sadness, fear, and anger/frustration), and Effortful Control, which shows self-regulatory abilities (inhibitory control, attention focusing). The mean scores for each subscale were computed by dividing each subscale’s total score by the number of items. The Cronbach’s alphas were 0.64 for Surgency/Extraversion, 0.70 for Negative Affectivity and 0.72 for Effortful Control. Similar coefficients were obtained by Putnam et al. (2010) on six samples of children 18–36 months of age: 0.72 for Surgency/Extraversion, 0.70 for Negative Affectivity and 0.72 for Effortful Control.

#### Delay of Gratification Task

Maternal regulatory attempts, toddlers’ self-regulatory strategies and toddlers’ emotional expressions were coded using a 4-min delay of gratification task, a reliable paradigm that was designed to measure children’s regulatory attempts during a waiting situation (Mischel et al., 1988). The task was videotaped with mother’s consent. Prior to the task, the toddler and mother were invited to sit at a table side-by-side, in a room with very few distractions. The experimenter placed a cookie on the table within the child’s reach and instructed the mother to complete a questionnaire and respond as she normally would, to make her child wait. The child was instructed to wait for the cookie until his/her mother finished her work. There were no other toys available for the child during this task. After making sure that both the mother and the child understood the instructions, the experimenter left the room for 4 min. The child received the cookie after the 4 min ended and the task was finished.

##### Maternal regulatory attempts

Maternal strategies were coded using a coding scheme developed by Corapci et al. (2013 unpublished). Based on the occurrences of corresponding behaviors, 12 maternal behavior codes (out of seventeen) were further used in the analysis. The maternal behavior codes were grouped in six main categories (based on previous literature, e.g., Grolnick et al., 1998). These broader categories were: (1) Warmth – Positive Emotional Reaction, Physical Comfort and Reassurance; (2) Expressive Encouragement; (3) Distraction; (4) Positive Control – Rule Statements, Prohibition Statements, Suggestive Commands and Reasoning; (5) Task Oriented Control – Removing Cookie and Refraining; and (6) Ignoring (Table 2 presents brief descriptions for all these codes). All videos were coded by two independent research assistants. 20% of the data were double coded in order to establish inter-rater agreement. Kappas ranged from 0.70 to 1.00. The presence or absence of each code was rated in 5-s epochs with multiple codes allowed in a given epoch. Proportions (i.e., percentage scores) were computed by dividing the number of epochs each specific code was rated as present, by the total number of epochs. The proportion scores were used in our analyses.

**Table 2:** Brief definitions of maternal and child regulatory attempts.

Maternal regulatory attempts codes	Description
**Warmth**	
Positive emotional reaction	Showing overt positive affect
Physical comfort	Hugging, kissing, stroking child’s hair
Reassurance	Providing reassurance to child
**Expressive encouragement**	Encouraging child to express emotions or validating, labeling emotions
**Distraction**	Shifting child’s attention by talking about non-task related topics, making suggestions for activities, pointing out to other objects
**Positive control**	
Rule statements	Statements such as “wait a minute, you can eat when the bell rings, sit down quietly”
Prohibition statements	Statements such as “don’t touch that, don’t scream now, you can’t leave now”
Suggestive commands	Suggestions, polite statements or questions (i.e., *Will you stop touching the cookie?)*
Reasoning	Explanations for compliance based on norms, values, or consequences. (i.e., “the lady told us not to touch this,” or “you have to wait because I have to fill out this important paper”)
**Task oriented control**	
Removing cookie	Removing cookie out of child’s sight or reach
Refraining	Stopping child’s action toward the cookie by holding back child’s arms
**Ignoring**	Not responding to child’s bids verbally or non-verbally within 3 s

**Child regulatory attempts codes**	**Description**

**Distraction**	
Child-initiated distraction	Child initiates or participates in alternative activities, shifts focus away from treat, begins an alternative behavior
Orienting to non-delay	Brief glances (about 1 s or so) on objects other than the cookie
**Orienting to delay**	Child focuses on the delay object
**Child self-comfort**	Physically or verbally comforts self; e.g., child hugs or pats self or sucks thumb
**Aggression**	
Behavioral aggression	Banging, venting, kicking, throwing, hitting the task object or aggression directed toward mother or experimenter.
Verbal aggression	Screaming, yelling, screeching
**Contact to mother**	
Child-initiated bids to engage mother	These are behaviors such as physical movement (e.g., tugging on mother) or vocal, verbal, or gestural that are meant to seek support or get mother’s attention while in an emotionally challenging situation.
Physical comfort seeking	Child seeks closeness to mother to be comforted

##### Toddlers’ regulatory attempts

Within the same 5-s epochs, a second team of coders examined toddlers’ regulatory attempts, based on the coding system developed by Corapci et al. (2013 unpublished). A total of eight codes, grouped in five main categories, inspired by similar approaches (e.g., Grolnick et al., 1996; Calkins and Dedmon, 2000) were used: (1) Distraction; (2) Orienting to Delay; (3) Child Self-Comfort; (4) Aggression, either behavioral or verbal; (5) Contact to Mother. Table 2 presents brief descriptions for all these codes. All videos were coded by other two independent coders. 20% of the data were double coded in order to establish inter-rater agreement. Kappas ranged from 0.70 to 0.90. The presence or absence of each code was rated in 5-s epochs with multiple codes allowed in a given epoch. Proportions (i.e., percentage scores) were computed by dividing the number of epochs each specific code was rated as present by the total number of epochs. The proportion scores were used in our analyses.

##### Toddlers’ anger, sadness, and happiness expressions

Previous research showed that toddlers quickly react with anger during delay tasks (Cole et al., 2011), but also display sadness (Buss and Kiel, 2004) and happiness (Dennis et al., 2009). Therefore, in the present study we coded the occurrence of these three emotions. Within the same 5-s epochs, a third team of coders rated the three basic emotions, based on toddlers’ facial expression, vocal tone, and postural characteristics, according to the coding scheme by Cole et al. (2007). Separate cues were used to identify the occurrence of each emotion on a 4-point scale (*0 = no sign of expression, 1*= *slight intensity, 2 = clear but moderate intensity, 3 = strong intensity*). If more than one emotion category was observed, each emotion was coded as present. Kappas were 0.85 for Anger, 0.80 for Sadness, and 0.90 for Happiness. The number of emotion-coded epochs was divided by the total number of epochs, to obtain proportion scores for each emotion. The proportion scores were used in our analyses.

For all coding schemes, the categories for which we encountered disagreements were discussed and more precisely described, by offering additional behavioral examples.

### Analytic Approach

In order to investigate whether mothers would equally use independent and interdependent self-descriptions (as expected from the emotional interdependence model), paired samples *t*-test was employed, for comparing Self-Construal Scale dimensions of Independence versus Interdependence, as well as the subdimensions of Autonomy/Assertiveness versus Relational Interdependence.

In order to analyze the relations between all maternal self-construal dimensions/subdimensions and maternal regulatory strategies (Warmth, Expressive Encouragement, Distraction, Positive Control, Task-Oriented Control and Ignoring) while controlling for the confounding effects of child gender and maternal education, we ran correlation analyses in the first step. Second, based on the predicted effects, according to our hypotheses, and the significant correlations obtained, we ran several multiple regression models, examining the effects of Individualism, Autonomy/Assertiveness, Primacy of Self and Relational Interdependence on maternal regulatory strategies, while controlling for maternal perception of child’s temperament.

Finally, in order to explore the associations between maternal regulatory strategies, child regulatory strategies (Distraction, Orienting to Delay, Self-Comfort, Aggression, Contact to Mother), and child affect (Anger, Sadness, and Happiness), during the delay of gratification task, we ran correlation analyses. Multiple regression analyses were further conducted, in order to assess whether child self-regulation mediates the relation between maternal regulatory strategies and child’s affect during the delay of gratification task.

## Results

### Maternal Self-Construal Dimensions

Means, standard deviations, and scale reliabilities, for all self-construal dimensions, are presented in Table 1. Paired samples *t*-test indicated a significant difference between maternal self-construal Independence versus Interdependence scores, *t*(39) = 2.75, *p* = 0.009. In addition, Autonomy/Assertiveness score was significantly higher than Relational Interdependence score, *t*(39) = 2.29, *p* = 0.03.

### Relations Between Maternal Self-Construal Dimensions and Maternal Regulatory Strategies

#### Correlation Analyses

First, we employed correlation analyses between maternal self-construal dimensions/subdimensions and maternal regulatory strategies (see Table 3). These analyses revealed that all subdimensions of Independence were associated with different maternal regulatory strategies. Individualism was negatively related to Physical Comfort (Warmth) and Removing Cookie (Task-Oriented Control) and positively associated with Ignoring. Autonomy/Assertiveness was also negatively associated with Removing Cookie (Task-Oriented Control). Primacy of Self was negatively associated with Positive Emotional Reaction (Warmth), but positively correlated with Expressive Encouragement and Prohibition Statements (Positive Control). Behavioral Consistency was also positively associated with Rule Statements (Positive Control). As for the Interdependence subdimensions, Relational Interdependence was positively correlated with Prohibition Statements (Positive Control).

**Table 3:** Pearson correlations between maternal self-construal and maternal strategies during the delay of gratification task.

	Maternal regulatory attempts											
	Positive	
Self-construal	emotional	Expressive	Physical			Rule	Suggestive			Prohibition	Removing	
subfactors	reaction	encouragement	comfort	Reassurance	Distraction	statements	commands	Refraining	Ignoring	statements	cookie	Reasoning
Esteem for group	0.07	0.13	–0.02	0.13	–0.02	0.08	0.00	0.05	–0.06	0.06	–0.21	0.00
Relational Interdependence	0.00	–0.15	–0.06	0.13	–0.17	0.24	0.17	–0.08	–0.07	0.32*	–0.25	0.04
Autonomy/ assertiveness	–0.23	0.16	–0.17	–0.09	0.10	0.11	0.06	–0.11	0.17	0.16	–0.34*	–0.12
Individualism	–0.23	0.06	–0.44**	–0.29	–0.04	0.02	0.03	–0.16	0.34*	0.09	–0.35*	–0.11
Behavioral consistency	0.04	0.03	–0.18	0.02	–0.14	0.31*	0.19	0.10	0.13	0.21	–0.27	0.07
Primacy of self	–0.31*	0.41*	0.03	–0.01	–0.13	0.14	0.07	0.02	0.09	0.33*	–0.05	0.06

*^∗^p ≤ 0.05, ^∗∗^p ≤ 0.01.*

In addition, in order to control the confounding effects of child gender and maternal education, we ran correlation analyses between these variables and maternal regulatory strategies. Child gender did not significantly correlate with any maternal regulatory strategy (*r*_s_ ranged from 0.00 to -0.30, *p* > 0.05), which ruled out its confounding influence. Only maternal education significantly and negatively (*r* = -0.41, *p* = 0.01) correlated with maternal Ignoring during the delay task. Therefore, maternal education was further considered in the regression model where Ignore strategy was the criterion.

#### Regression Analyses

Second, based on the predicted effects and significant associations between maternal self-construal dimensions/subdimensions and maternal regulatory strategies during the delay of gratification task, several regression models were conducted. This analysis explored the relations between maternal self-construal and maternal regulatory strategies, while also controlling for maternal perception of child temperament, given its significant associations with several maternal self-construal dimensions/subdimensions (see Table 4). For all these regression models, variables were entered in the following order: temperament (Surgency, Negative Affectivity and Effortful Control) in the first step, and maternal self-construal in the second step. Given our moderate sample size, in these regression models we examined only those dimensions/subdimensions of the Self-Construal Scale that demonstrated significant correlations with our criterion variables (maternal regulatory strategies) (see the above results on correlation analyses).

**Table 4:** Pearson correlations between maternal self-construal and maternal perception of child temperament.

	Maternal perception of toddler temperament		
Maternal self-construal	Negative		Effortful
dimensions	affectivity	Surgency	control
Independence	–0.25	0.45**	0.36*
Interdependence	0.17	0.18	0.06
Autonomy/assertiveness	–0.08	0.22	0.16
Individualism	–0.19	0.33*	0.17
Behavioral consistency	–0.26	0.36*	0.35*
Primacy of self	–0.12	0.34*	0.17
Esteem for group	0.10	0.23	0.22
Relational interdependence	0.06	0.17	0.02

*^∗^p ≤ 0.05, ^∗∗^p ≤ 0.01.*

Therefore, the first model examined the effect of Individualism on Physical Comfort (Warmth) (see Table 5). Results revealed that higher levels of maternal Individualism predicted lower use of Physical Comfort during the delay of gratification task [β = -0.45, *t*(39) = -2.90, *p* = 0.006]. The second model examined the effect of Autonomy/Assertiveness on Removing Cookie (Task-Oriented control). Autonomy/Assertiveness did not predict Removing Cookie after controlling for child’s temperament [β = -0.30, *t*(39) = -1.94, *p* = 0.06]. However, the third model (see Table 5), for the effect of Individualism on Removing Cookie, showed that Individualism predicted lower use of Removing Cookie [β = -0.36, *t*(39) = -2.26, *p* = 0.03]. Fourth, although not predicted in our hypotheses, we examined the effect of maternal Individualism on Ignoring strategy while controlling for maternal education (see Table 5). This analysis showed that higher levels of maternal Individualism did not significantly predicted higher levels of Ignore during the delay of gratification task [β = 0.32, *t*(39) = 1.98, *p* = 0.06] after controlling for maternal education [β = -0.45, *t*(39) = -2.82, *p* = 0.008]. The fifth model examined the effect of Primacy of Self on Expressive Encouragement (see Table 6) and it revealed that maternal Primacy of Self was a significant and positive predictor of Expressive Encouragement [β = 0.49, *t*(39) = 3.35, *p* = 0.002]. However, Primacy of Self did not predict maternal Positive Emotional Reaction (Warmth) after controlling for child’s temperament [β = -0.31, *t*(39) = -1.93, *p* = 0.06]. Finally, the model which tested the impact of Relational Interdependence on Prohibition Statements (Positive Control) revealed no significant effects [β = 0.29, *t*(39) = 1.77, *p* = 0.09].

**Table 5:** Summary of hierarchical regression analyses for maternal self-construal subdimension of Individualism predicting maternal regulatory strategies.

Predictor	Δ*R*^2^	*SE b*	β
Individualism, maternal perception of toddler temperament → Physical comfort			
**Step 1**	0.09		
Negative affectivity		2.74	–0.13
Surgency		3.28	–0.06
Effortful control		3.66	–0.31
**Step 2**	0.18		
Individualism		3.11	–0.45**
Total *R*^2^	0.27		
Individualism, maternal perception of toddler temperament → Removing cookie			
**Step 1**	0.11		
Negative affectivity		1.10	–0.28
Surgency		1.28	–0.12
Effortful control		1.42	–0.27
**Step 2**	0.12		
Individualism		1.26	–0.36*
Total *R*^2^	0.23		
Individualism, maternal perception of toddler temperament → Ignoring			
**Step 1**	0.19		
Negative affectivity		3.69	0.07
Surgency		4.42	–0.05
Effortful control		4.92	0.04
Maternal education		3.98	–0.45*
**Step 2**	0.09		
Individualism		4.15	0.32
Total *R*^2^	0.28		

*^∗^p ≤ 0.05, ^∗∗^p ≤ 0.01.*

**Table 6:** Summary of hierarchical regression analyses for maternal self-construal subdimension of Primacy of Self predicting maternal Expressive encouragement.

Predictor	Δ*R*^2^	*SE b*	β
Primacy of Self, maternal perception of toddler temperament → Expressive encouragement			
**Step 1**	0.13		
Negative affectivity		0.52	0.28
Surgency		0.62	–0.24
Effortful control		0.69	0.40
**Step 2**	0.22		
Primacy of self		0.41	0.49**
Total *R*^2^	0.35		

*^∗^p ≤ 0.05, ^∗∗^p ≤ 0.01.*

### Associations Between Maternal Regulatory Strategies, Child Regulatory Strategies and Child Affect During the Delay of Gratification Task

#### Correlation Analyses

The correlations between maternal regulatory strategies and child regulatory strategies are provided in Table 7. Results revealed that specific maternal Task-Oriented Control strategies (i.e., Removing Cookie) and specific maternal Positive Control strategies (i.e., Rule Statements) were significantly and negatively associated with child-initiated Distraction. In addition, maternal Positive Control in the form of Suggestive Commands was positively associated with child Physical Comfort Seeking and Behavioral Aggression, while maternal Positive Control in the form of Prohibition Statements was also positively associated with child Physical Comfort Seeking. Also, the maternal Task-Oriented Control strategy of Refraining was positively associated with child Behavioral Aggression and Physical Comfort Seeking. Maternal Warmth strategies correlated significantly with child Aggression and child Physical Comfort Seeking. Specifically, maternal Physical Comfort positively correlated with child Verbal and Behavioral Aggression, maternal Reassurance was positively correlated with child Physical Comfort Seeking and Behavioral Aggression, respectively, while Reasoning was positively associated with Physical Comfort Seeking and Behavioral Aggression. Finally, we found that maternal Expressive Encouragement as well as Ignoring were positively associated with child Verbal Aggression and Contact to Mom.

**Table 7:** Pearson correlations between maternal regulatory attempts, child regulatory attempts and child affect during the delay of gratification task.

	Child regulatory attempts								Toddler’s affect		
Maternal regulatory	Child-initiated	Orienting	Orienting	Physical comfort	Child	Behavioral	Verbal	Contact			
attempts	distraction	to non-delay	to delay	seeking	self-comfort	aggression	aggression	to mother	Anger	Sadness	Happiness
Positive emotional reaction	0.20	–0.16	–0.05	–0.12	0.27	–0.10	–0.19	0.03	–0.30*	–0.46*	0.61**
Expressive encouragement	–0.11	–0.01	–0.02	–0.15	–0.05	–0.16	0.33*	0.43**	0.06	0.30	0.28
Physical comfort	–0.22	0.08	0.05	–0.03	0.01	0.42**	0.37*	–0.09	0.50**	–0.05	–0.17
Reassurance	–0.13	0.01	0.03	0.37*	0.08	0.48**	–0.01	0.11	0.23	–0.15	–0.12
Distraction	0.22	0.01	0.00	–0.22	0.21	0.04	–0.23	–0.03	0.10	–0.16	–0.06
Returning attention	–0.21	–0.05	–0.01	–0.09	–0.09	–0.14	–0.07	0.11	0.21	0.05	0.09
Rule statements	–0.62**	0.03	–0.01	0.05	0.00	0.17	0.09	0.20	0.56**	0.03	–0.17
Suggestive commands	0.29	–0.21	–0.23	0.77**	–0.09	0.48**	–0.05	–0.09	–0.13	0.05	–0.09
Refraining	0.14	–0.14	–0.13	0.31*	–0.10	0.35*	0.21	–0.12	0.69**	0.28	–0.12
Ignoring	–0.07	–0.01	–0.03	–0.10	–0.05	–0.10	0.50**	0.34*	0.15	0.65**	0.16
Prohibition statements	–0.13	0.05	0.02	0.38*	–0.16	0.24	–0.02	0.20	0.07	–0.01	–0.16
Reasoning	0.09	–0.14	–0.08	0.54**	0.07	0.53**	–0.08	0.09	0.02	–0.11	–0.03
Removing cookie	–0.54**	0.21	0.20	–0.14	–0.10	0.27	–0.10	–0.19	0.73**	0.24	–0.13

*^∗^p ≤ 0.05, ^∗∗^p ≤ 0.01.*

Regarding child affect during the delay task, maternal Positive Emotional Reaction was significantly associated with less Anger and Sadness, but with more positive affect (Happiness). Moreover, Physical Comfort, Refraining, Removing Cookie, and Rule Statements were also associated with more Anger, while Ignore was associated with more Sadness. We also examined the relation between child regulatory strategies and child affect. In this respect, we found that child-initiated Distraction was associated with significantly less Anger, *r*(39) = -0.61, *p*= 0.001 and less Sadness, *r*(39) = -0.41, *p* = 0.03. In addition, more Anger was associated with the use of Behavioral, *r*(39) = 0.31, *p* = 0.04 and Verbal Aggression, *r*(39) = 0.39, *p* = 0.04, and more Sadness with Verbal Aggression *r*(39) = 0.52, *p* = 0.001.

#### Regression Analyses

**FIGURE 1:**
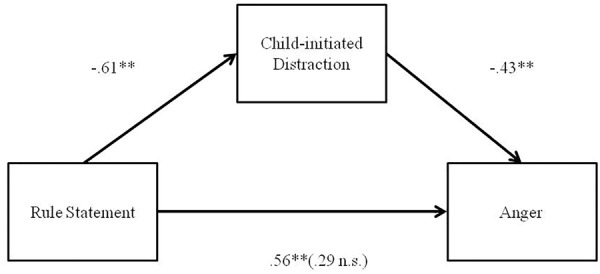
Indirect effect of maternal Rule Statement on Child Anger through child-initiated Distraction. ^∗^*p* < 0.05, ^∗∗^*p* < 0.01.

Multiple regression analyses were conducted in order to assess whether child self-regulation mediates the relation between maternal regulatory strategies and child’s affect during the delay of gratification task. The model that fulfilled all criteria for mediation testing was the one in which child-initiated Distraction was considered a mediator between maternal Rule Statement (Positive Control) and child expression of Anger. Results demonstrated that maternal Rule Statement strategy was positively associated with child Anger [β = 0.56, *t*(39) = 4.20, *p* = 0.000]. It was also found that maternal Rule Statement was negatively related to child-initiated Distraction [β = -0.61, *t*(39) = -4.82, *p* = 0.000]. Lastly, results indicated that the mediator, child-initiated Distraction, was negatively associated with child Anger [β = -0.43, *t*(39) = -2.74, *p* = 0.009]. Because both the a-path and b-path were significant, mediation analyses were tested using the bootstrapping method with bias-corrected confidence estimates (MacKinnon et al., 2004; Preacher and Hayes, 2004). In the present study, the 95% confidence interval of the indirect effects was obtained with 5000 bootstrap resamples (Preacher and Hayes, 2008). Results of the mediation analysis confirmed the mediating role of child Distraction for the relation between maternal Rule Statement and child Anger (b = -0.43, 95% CI [0.05, 1.17]). In addition, results indicated that the direct effect of maternal Rule Statement strategy on child Anger became non-significant [β = 0.29, *t*(39) = 1.89, *p* = 0.06] when controlling for child-initiated Distraction, thus suggesting full mediation. Figure 1 displays the results.

## Discussion

The present paper circumscribes the socialization of emotion in toddlerhood, exploring maternal regulatory attempts on child emotional expressions, displayed within a delay of gratification task. Given that emotion regulation is not only socially but also culturally embedded, we were interested in analyzing maternal regulatory behaviors in relationship to maternal cultural models of self and relating (De Leersnyder et al., 2013; Mesquita et al., 2014; Jaramillo et al., 2017).

We focused on a specific cultural context, the Romanian context, which, given the sociodemographic changes over the last decades, stands outside the standard individualism versus collectivism dichotomy. The ambivalence individualism-collectivism at the societal level (Gavreliuc, 2011) expressed in a decrease in collectivistic socialization values and a corresponding increase in individualistic ones (Greenfield, 2009), is presumably reflected in the emotionally interdependent (combined independent-interdependent) individual self-construal of middle-class educated Romanians (Mone et al., 2014; Moza et al., 2015; Corapci et al., 2017). However, the independence-interdependence balance in self-construal is not rigidly set, being therefore worthy of further exploration, particularly in relationship with child emotion socialization.

The goals of this study were twofold: (1) to examine, in a Romanian sample of mother-toddler dyads, the relationships between maternal cultural model of the self and maternal regulatory attempts targeting toddlers’ emotions, during a delay of gratification task, while controlling for maternal perceptions of child individual characteristics (namely, temperament), child gender and maternal education; (2) to analyze, within the delay of gratification task, the relations between maternal regulatory attempts, child regulatory strategies, and child affect expression as the outcome of emotion regulation.

### Maternal Cultural Model of the Self and Maternal Regulatory Attempts on Toddler’s Emotions

#### Emotional Interdependence or (a Shift Toward) Independence?

Grounded on the rather limited existing literature, dedicated to this topic and to the cultural niche of interest, respectively, we expected that Romanian mothers will experience a balanced view of an independent and an interdependent self, thus a view congruent with the emotionally interdependent self model (Kagitcibasi, 2007). However, yet in consonance with the results of Gavreliuc and Ciobotă (2013), Mone et al. (2014), Moza (2017) on larger samples, mothers from our sample scored higher for Independence, as compared to Interdependence, in self-construal measurements. Also, the multidimensional analysis of self-construal that allowed for comparisons between several subdimensions of the self, further revealed that Autonomy/Assertiveness scores were significantly higher than Relational Interdependent scores.

This implies that, in our sample of urban, educated, middle-class Romanian mothers, there is a shift toward an independent representation of the self and its relationships with the world. This weight put on individuality may well be an adjustment to the pressures of a highly competitive environment, which has become characteristic, in the last decades, for Romanian urban settings like Cluj-Napoca (the second most populous city in Romania, and one of the most important business and academic centers in the country). Given the age of mothers in our sample (mean = 31.5 years, *SD* = 3.2 years), we can say they grew up along with the post-communist socio-economic changes, from the very onset of these transformations in the early 90s, and highly likely have internalized, to a considerable degree, the ideal representation of self, brought along by this shift toward market economy, globalization and urbanization. Also, their high levels of education and employment speak about their commitment to the challenges and rewards of this societal setup. This speculative interpretation of our results deserves further investigation.

In the same time, we must acknowledge, along with Markus and Kitayama (1991) and Vignoles et al. (2016), that different cultures foster different ways of being independent, and interdependent, respectively. Therefore, a preference for an independent self-construal may have different meanings, as well as different corresponding behaviors, for someone from our sample, as compared to someone from an independence-promoting culture, like United States. Congruently with this idea, Moza and Gavreliuc (2017 unpublished) found that, for Romanian young adults, different facets of independence in the self-construal, i.e., viewing themselves as being consistent across contexts and groups, being self-reliant, and being able to express their self when communicating with others, were associated with higher self-esteem and happiness. On the other hand, for the American young adults, consistency across contexts and groups, and seeing themselves as different from others, were the subdimensions of independence associated with higher self-esteem. Future studies on the self-construal of Romanian parents will have to address this issue of multidimensionality and specific meanings of a certain self view.

#### Maternal Self-Construal and Social Regulation of Emotion by Maternal Attempts

Under the first aim of the study, we explored the relationships between maternal cultural model of the self and maternal regulation of toddlers’ emotions, during a delay of gratification task. Based on previous literature (Jaramillo et al., 2017), we expected an emphasis on Independence to be negatively associated with Task-Oriented Control strategies, and positively with a stress on agency and engagement of the child in goal-directed behaviors, e.g., via Distraction.

Our multifaceted analysis of the Self-Construal and, in particular, of the construct of Independence (in terms of Autonomy/Assertiveness, Individualism, Behavioral Consistency, and Primacy of Self), in relationship to maternal regulatory strategies, revealed that mothers who consider themselves higher in this respect give space to their children, both in terms of behavioral self-regulation and of emotion expression/regulation. They do not seem to feel their relationship with the child is jeopardized because of her/his anger and distress, nor act to calm down the child quickly. Indeed, an independent model of the self (as the one evidenced to be augmented in our sample), is expected to have consequences in terms of defining what a “good” parent–child relationship means: namely, one that allows for full expression of autonomy and individuality, even in situations of “crisis,” like that prompted by the delay of gratification task, when the child is more likely to express disengaging emotions, e.g., anger or dissatisfaction (see; Heikamp et al., 2013; Jaramillo et al., 2017).

Different subdimensions of Independence predicted different uses of maternal regulatory strategies. Mothers high in Individualism, who enjoyed their uniqueness, were more likely to: (a) maintain physical boundaries between them and their children, instead of relying on soothing, when faced with their children’s distress (low Warmth by Physical Comfort); (b) leave room for child self-regulation (low Task-Oriented Control by Removing Cookie). Thus, high Individualism predicted a decreased use of maternal Warmth and Task-Oriented Control. These strategies are similar to the ones displayed by mothers from independence-promoting cultures, as documented by other studies on parent-infant and parent-toddler interaction, respectively (e.g., less physical interaction and soothing, less directiveness, more space and support for child initiatives – see Keller et al., 2006; Corapci et al., 2017; Lamm et al., 2017). The added value of the study resides in showing that the same strategies are present in the behavioral repertoire of mothers from an emotionally interdependent culture, being associated with the emphasis on an independent view of the self such mothers might have.

In addition, mothers high in pursuing their own good and interests, irrespective of the others (Primacy of Self), encouraged and validated to a higher extent their children’s emotions (Expressive Encouragement), but were not prone to show positive affect (Positive Emotional Reaction), possibly in a situation rather uncomfortable for themselves as well. In other words, an augmented Primacy of Self in the Self-Construal predicted less maternal Warmth, but more maternal Expressive Encouragement in the delay of gratification task.

Our results partially confirmed our predictions, since no positive associations were found between Independence (and/or its subdimensions) and the maternal strategy of Distraction. Also, our hypothesis regarding the positive relationships between Relational Interdependence, Warmth and Positive Control, respectively, was not confirmed.

Taken together, our data suggest the matching between an independent view of the self and maternal strategies that foster autonomy and self-regulation in the child. In other words, maternal representations of an independent self, as evidenced in our sample, were mirrored into regulatory practices aimed to develop primary control in the toddler, meaning autonomy, assertiveness and self-realization, stemming from the belief that the world must change according to the needs of the (fixed) self (Rothbaum and Wang, 2010; Jaramillo et al., 2017). This is one of the very few studies addressing the direct relationship between parental self-conceptualizations and parental behavioral variables. Thus, our data bring valuable and distinctive evidence on the relationship between a subjective measure of the self-construal and an observational measure of maternal regulatory practices with toddlers, as displayed in an emotionally charged context.

#### Maternal Perception of Toddler Temperament – A Potentially Confounding Variable

We also controlled for maternal perceptions of child individual characteristics, namely, temperament. In this respect, our data showed that different subdimensions of the self-construal were also associated with maternal perceptions of child characteristics. Individualism, Behavioral Consistency and Primacy of Self were associated with higher perception of child Surgency. This finding is in line with other data, showing that Surgency is positively associated with Hofstede’s individualism parameter and is higher in individualistic, as compared to collectivistic countries (Putnam and Gartstein, 2017). Also, the tendency of and the “tolerance” for the experience and expression of high-activation, high intensity emotions in individualistic cultures (Matsumoto et al., 2008), is congruent with this finding. On the other hand, high Behavioral Consistency and Independence were associated with perceptions of temperamental Effortful Control. This speaks about the encouragement of self-regulation, probably understood as motivated, autonomous action (Trommsdorff, 2009), by mothers in our sample.

Maternal regulatory attempts were, at their turn, influenced by maternal perceptions of child characteristics. While high maternal Autonomy/Assertiveness reduced maternal tendency to change the environment in order to lessen child discomfort (via Removing Cookie), its predictive value was nulled after controlling for maternal perceptions of child temperament (in particular Negative Affectivity). This suggests social regulation of emotion is not independent of child characteristics, but rather calibrated to them, as also demonstrated by previous research (Kochanska et al., 2007).

### Maternal Regulatory Attempts, Child Regulatory Strategies and Child Affect Expression

The second aim of our study was to analyze, within the delay of gratification task, the relations between maternal regulatory attempts, child regulatory strategies, and child affect expression.

Our data revealed several significant associations between maternal regulatory strategies and child regulatory strategies expressed during the delay of gratification task. Maternal Warmth (Reassurance) and Positive Control (Reasoning) strategies were positively related to toddlers’ Contact to Mother (Physical Comfort Seeking), but also to Behavioral Aggression. Also, both a Positive Control strategy (Rule Statements) and a Task-Oriented Control strategy (Removing Cookie), as non-distracting strategies, were associated with less Child-Initiated Distraction. Moreover, less supportive maternal strategies, such as Ignoring and Task-Oriented Control (by Refraining) were positively related to toddlers’ Verbal Aggression, Contact to Mother (Physical Comfort Seeking) and Behavioral Aggression. Mother’s encouragement of children to express negative/positive affect, or validation of the child’s negative/positive emotional states (Expressive Encouragement), was associated with higher child Verbal Aggression and Contact to Mother, while maternal Warmth (Physical Comfort) with higher Verbal and Behavioral Aggression.

The relations between mothers’ use of non-distracting, in particular Warmth and Task-Control strategies, and child’s Aggression, as well as Contact to Mother, are in line with previous research, showing that mothers who use less attention shifting behaviors (like redirecting children’s attention or providing comfort) have toddlers who use to a higher degree less efficient emotion regulation strategies, in terms of modulating emotional arousal (Mirabile et al., 2009). It is noticeable that the same directions of associations were found in these studies performed on United States samples, coming from parents with a presumable (since not measured) independence-promoting orientation.

Also, in our sample, toddlers who used more Behavioral and Verbal Aggression expressed more negative emotional reactions, such as Anger and Sadness. The association between mothers’ Expressive Encouragement and children’s aggression can be interpreted through the lenses of the increased expression of negative disengaging emotions and of their behavioral manifestations, as a consequence of maternal belief in expressing oneself’ as an autonomous individual, irrespective of the social consequences (Heikamp et al., 2013). In other words, these data also support the idea of maternal practices that foster primary control in toddlers from our sample. On the other hand, previous data with toddlers also demonstrated that maternal supportive or distractive strategies, such as verbal distraction, were associated with children’s aggression or children’s angry outbursts (Garner and Spears, 2000; Mirabile et al., 2009). Thus, during this developmental period, aggressive acts may emerge when toddlers are overwhelmed by a distressing situation, even under optimal socialization conditions.

The current data from our sample point toward a coherent pattern of representation and actions (including parental regulatory practices) matching the independence model of self. Still, we favor the perspective of multiple facets of emotional interdependence, which can, under certain conditions, incline the balance beam toward independence. This interpretation is based not only on Kagitcibasi’s theoretical model (2007), and on previous results with other, different, Romanian samples, but also on our own data, explored from the perspective of most prevalent maternal regulatory strategies. In this sense, mothers from our sample employed Distraction, Ignoring, Positive Rule Statement, and Physical Comfort as first four choices, that is, a mixture of independence- and interdependence-promoting strategies.

Within the developmental framework of social regulation of emotion in toddlerhood (Kopp, 1989; Reeck et al., 2016), we assessed, via multiple regression analyses, whether child self-regulation mediated the relation between maternal regulatory strategies and child’s affect during the delay of gratification task. The only model that fulfilled all criteria for testing mediation was the one in which child-initiated Distraction was considered a mediator between maternal Rule Statement and child expression of anger. Results suggest a full mediation of the relation between maternal Rule Statement and child anger by child Distraction. This analysis provides one, out of the few existing examples, of the role played by the socialization of emotion on the emotional expression, by child self-regulatory strategies. They underline the idea that parental emotion regulatory strategies foster child self-regulatory strategies, which further impact on emotion.

### Limitations

Results presented here are, of course, subject to several limitations. First, we explored regulatory attempts of mother and child, as well as child’s emotional expressions solely within a laboratory task; thus results cannot be generalized to more ecological settings and have a rather limited explanatory power, as concerning daily parent–child interaction dynamics. Complementary methods could bring more light regarding this issue in future studies. Second, the high proportion of educated, employed, and middle-class mothers from our sample limits the generalizability of our results to the majority of Romanian population. Yet, predictions of Kagitcibasi’s theory (2007) on autonomous-relatedness specifically target this demographic segment. However, since economic, social and political change may differentially impact cultural values and prevalent parental beliefs and practices in people with lower SES or education levels, a more heterogenous sample would be more informative. Third, our sample was restricted to mothers, thus our results may be limited regarding father-toddler regulatory dynamics. Fourth, our sample size did not ensure the statistical power to test within a single multi-variable model the relation between maternal self-construal dimensions, maternal regulatory strategies, and child regulatory attempts. Also, the null findings should be interpreted with caution, as our sample was not large enough. Fifth, the internal consistencies of two self-construal subscales were lower than desired, though in line with past research (Hardin et al., 2004). Sixth, evidence provided by this study is limited, due to its cross-sectional design. Finally, we did not have measures of socialization goals and parental ethnotheories that would have offered a more complete landscape of the maternal cultural model.

## Conclusion

Given these limitations, the current study still adds to the existing knowledge some significant insights regarding the impact of maternal self-construal on social regulation of emotion, by means of maternal behaviors, in toddlerhood – a critical age for the development of self-regulation. Within the boundaries of a correlational design, it brings valuable data on the relationship between a subjective measure of maternal self-construal and an observational measure of maternal regulatory strategies with toddlers. As a culturally permeated variable, self-construal is sensitive to the cultural orientation of a society, that is assumed to be further affected by socio-demographic changes. Thus, studying its interplay with parenting regulatory strategies, in a cultural context undergoing significant societal changes in the last decades, offers relevant data for testing theories of culture and family change.

The current study also explores the anatomy of maternal regulatory behaviors and their association with the emerging child regulation strategies, the implications of such exploration extending beyond the particular cultural context in focus. This fine-grained analysis enhances the understanding of the mechanisms involved in toddler self-regulation development, in relation with the (adult-to-child) social regulation of emotion. Last but not least, it informs about the dynamics of these variables in a less studied cultural context, the Romanian one, providing an interesting example of proneness to independence, within an emotionally interdependent model of the self.

Findings like the present ones could guide more nuanced views on parenting strategies and parenting programs, that are critical for enhancing parental skills during the “terrible twos” – a normative developmental stage in which toddlers begin to struggle between their reliance on adults and their desire for independence. Most programs are currently imported from Western cultural contexts, under the influence of globalization, with little or no adaptation to cultural idiosyncrasies. Therefore, our findings could inform and focus parenting programs on culturally sensitive strategies, for example, by providing a mixture of independence- and interdependence-promoting strategies that guide and support child self-regulation (in cultures like the Romanian one).

## Ethics Statement

This study was carried out in accordance with the recommendations of Ethics Committee of Babes-Bolyai University and by requesting written informed consent from all participants.

## Author Contributions

OB contributed to study design, data collection and processing, statistical analysis, interpretation, paper writing and review. GS-E contributed to data collection and processing, statistical analysis, interpretation, paper writing and review. WF and FC contributed to study design, supervision of data coding training, paper writing. MR contributed to data collection, processing, statistical analysis, and paper writing.

## Conflict of Interest Statement

The authors declare that the research was conducted in the absence of any commercial or financial relationships that could be construed as a potential conflict of interest.
